# Error Reduction in Healthcare Through Team Training and Cultural Transformation

**DOI:** 10.7759/cureus.91243

**Published:** 2025-08-29

**Authors:** Mohammadali M Shoja, Darisel N Ventura Rodriguez, Olga Avilova, Vijay Rajput

**Affiliations:** 1 Medical Education, Nova Southeastern University Dr. Kiran C. Patel College of Allopathic Medicine, Fort Lauderdale, USA; 2 General Surgery, Nova Southeastern University Dr. Kiran C. Patel College of Allopathic Medicine, Fort Lauderdale, USA

**Keywords:** artificial intelligence, error management, healthcare policies, medical education, medical errors, patient safety

## Abstract

Despite major advancements in healthcare delivery, preventable medical errors remain a significant public health concern, and are a leading cause of death in the United States. These errors are often rooted in communication failures, a lack of effective teamwork, and rigid hierarchical structures within healthcare teams. This persistent problem highlights the urgent need for a paradigm shift in how care is delivered, one that prioritizes a collaborative, system-based approach over individual blame.

The aim of this paper is to emphasize the critical need for a paradigm shift in healthcare, focusing on teamwork, communication, and leadership as essential components of patient safety and quality of care. Drawing inspiration from the aviation industry's Crew Resource Management (CRM) model, a team training framework that emphasizes communication, decision-making, and situational awareness to reduce human error, this paper highlights the evolution of CRM, which has culminated in a comprehensive approach encompassing threat and error management. This aviation model serves as a compelling role model for healthcare, illustrating the necessity of improving collaborative practices among healthcare professionals.

The paper also touches on the applicability of Reason's Swiss Cheese Model of System Accidents, a conceptual framework illustrating how errors occur when multiple layers of system defenses fail simultaneously, as a robust theoretical foundation for team training programs in healthcare. The authors emphasize the importance of replacing a culture of blame with one of mutual respect and shared responsibility in healthcare, and advocate for the integration of approachability training and the promotion of psychological safety in the workplace.

To bridge communication gaps in healthcare, the authors advocate for more extensive interprofessional training programs to foster effective teamwork from the earliest stages of professional training and throughout one’s career. The paper concludes that the next healthcare revolution should prioritize teamwork, communication, and leadership to ensure patient-centered, safe care by leveraging artificial intelligence (AI) and large language models (LLMs), a class of AI tools capable of analyzing and generating human-like language, to support these goals.

## Introduction and background

As David A. Marshall aptly noted, “A system not designed to expect and safely absorb human error will constantly suffer from human mistakes” [1].

The aviation and healthcare industries are both considered high-reliability organizations with a generally low probability of mishaps, as noted by Speers [1]. However, the aviation disasters of the 1970s and 1980s highlighted that communication breakdowns and team errors were major contributing factors. The urgency of the situation and public outcry compelled the aviation industry to make significant changes to its communication protocols. This led to the emergence of the “Crew Resource Management” (CRM) program and a new culture of teamwork that replaced the traditional hierarchical leadership [2]. CRM refers to a structured system of communication and teamwork strategies originally developed in aviation to reduce errors. CRM training became mandatory for the US civil aviation industry in 1998, as reported by Marshall [3]. Though articulated more than a decade ago, the assertion by Gordon and colleagues remains strikingly pertinent today: in terms of safety protocols and team-based training, the healthcare system continues to operate in a fragmented, hierarchical, and siloed manner, much like the aviation industry in the 1970s and 1980s, before the implementation of CRM. At that time, aviation faced high rates of preventable accidents due to poor cockpit communication, rigid authority structures, and a lack of coordinated training, challenges that still resonate in modern healthcare environments, particularly in high-stakes settings such as emergency departments, operating rooms, and intensive care units. Since the 1990s, several medical team training programs inspired by the aviation industry have been developed with the goal of improving healthcare teamwork, reducing errors, and enhancing patient safety [4].

The review examines the evolution of aviation CRM, the persistent patient safety crisis in healthcare, and key similarities and differences between the two industries. Grounded in Reason’s Swiss Cheese Model, it advocates for a system-based approach to medical errors. The Swiss Cheese Model is a widely used metaphor for error prevention, illustrating how multiple layers of defense can align to allow mistakes to occur [4]. It reviews CRM-inspired team training in healthcare, outlining core concepts, objectives, and future directions, including the emerging role of AI and large language models (LLMs) in enhancing team training.

## Review

Evolution of CRM in aviation

CRM originated in the late 1970s, following a series of aviation disasters where poor communication and hierarchical cockpit dynamics were identified as major contributing factors. CRM in aviation aims to “organize a group of individuals to think and act as a team with the common goal of safety,” as noted by McConaughey [5]. As Baker and colleagues have pointed out, “teamwork” is not an automatic consequence of placing people together but a skill that necessitates interdisciplinary training [6]. Since its inception, aviation CRM has undergone continuous modification and updates (Table 1), evolving from a narrow focus on cockpit communication to a comprehensive approach that includes error and threat management across the entire flight team.

**Table 1:** Evolution of CRM in aviation CRM: Crew resource management

Generation	Description
1^st^	Focused on flight deck communication with the crew.
2^nd^	Emphasized teamwork, situational awareness, communication, and stress/workload management.
3^rd^	Extended training to all flight members, who were jointly trained.
4^th^	Integrated CRM into all flight training.
5^th^	Included an error management approach.
6^th^	Emphasized threat and error management.

The scale of preventable medical errors in healthcare

In 1999, the Institute of Medicine (IOM) published a report estimating that approximately 100,000 patients die each year in the United States (US) due to preventable medical errors in hospitals [7]. The IOM’s estimate was based on data from 1984, and subsequent analyses have suggested that this figure significantly underestimates the current situation [8]. Swartz made a striking comparison, stating that the annual deaths from preventable medical errors are “equivalent to losing passengers on two jumbo jet crashes per day” or 1,200 patients daily [8]. Nearly more than two decades following the IOM report, Makary and Daniel noted the death toll attributable to medical errors had doubled, surpassing 200,000 [9]. This positioned medical errors as the third leading cause of death in the US, trailing only heart disease and cancer. Recognizing the potential for under-representation, the recent studies have called for enhanced reporting systems and improved documentation in death certificates to more accurately reflect the incidence of medical errors [10,11]. This sobering data underscores the gravity of the issue. Speers and McCulloch further contend that with such a high error rate, the healthcare system in North America cannot be classified as a highly reliable organization [1].

Similarities and differences between the aviation and healthcare industries

While there are notable functional and managerial parallels between the aviation and healthcare industries, it is also crucial to acknowledge the significant differences between the two (Table 2).

**Table 2:** The similarities and differences between aviation and healthcare industries

Similarities	Differences
High-reliability, safety-critical industries	In aviation, failure often brings about mass casualties, while in healthcare, failure impacts one patient at a time.
Use of high technology	
High risk	
High cost of errors	In aviation, there is an overall uniformity of the flight operation, while each patient requires an individualized plan.
Redundancy in operations	
High-stress and complex environment	
High workload	Healthcare is more complex and ever-changing.
Work under multiple, standardized guidelines	

As Grumet rightly pointed out, “...human beings, especially, do not follow a manual. The number of interconnecting parts is too great” [12]. Aviation systems are structured and predictable, while the human body is complex, adaptive, and harder to standardize. This makes it challenging to apply aviation-based training models directly to healthcare.

Person versus system approach to human error in healthcare

Reason has delineated two distinct approaches to addressing the issue of human error [13]. The first approach, the person approach, is characterized by a culture of blame and seeks to isolate an individual’s harmful actions from institutional responsibility. In contrast, the system approach promotes a culture of trust and encourages unbiased reporting. Reason argues that blaming individuals prevents progress in patient safety. He supports the system approach, which focuses on managing risks before harm occurs.

In Reason’s view, hidden problems in the system, often caused by poor decisions from leaders, create conditions for error. These latent conditions are typically dormant until they combine with active failures of individuals directly interacting with patients, often the distal elements of the system. When latent conditions and active failures align, accidents can occur, jeopardizing patient safety. For example, a hospital may have a latent condition such as a poorly designed electronic medical record (EMR) system that makes medication orders difficult to review accurately. If, on a particularly busy shift, a fatigued physician overlooks a drug interaction warning (an active failure), the combination of these two factors may result in a harmful medication error. 

Reason’s Swiss cheese model of system accidents provides a strong theoretical foundation for team training programs in healthcare by emphasizing how errors arise not solely from individual actions, but from systemic vulnerabilities that require team-based solutions. In addition to Reason’s Swiss cheese model, the Software-Hardware-Environment-Liveware (SHEL) framework provides a complementary and insightful approach to understanding error in healthcare systems [13]. The SHEL model focuses on how these components interact, emphasizing that safety depends on the quality of their integration. Molloy and O’Boyle emphasize that this model is particularly valuable in high-risk healthcare environments, such as emergency rooms and operating rooms, where rapid decision-making, heavy reliance on technology, and complex interpersonal interactions heighten the risk of errors and safety breakdowns. They highlight that mismatches between software (e.g., protocols or EMRs), hardware (e.g., equipment), environmental factors (e.g., noise, lighting), and liveware (e.g., human capabilities and limitations) often result in adverse outcomes when these elements are not well aligned [14].

From our perspective, the SHEL model can guide healthcare institutions in identifying gaps in communication and system design. For example, regular simulation-based training can help assess how team members interact with their tools and environments, revealing hidden risks and informing targeted interventions. By examining errors through the lens of the SHEL framework, healthcare leaders can move beyond individual blame and implement more holistic safety strategies.

Reason’s Swiss cheese model of system accidents

**Figure 1:**
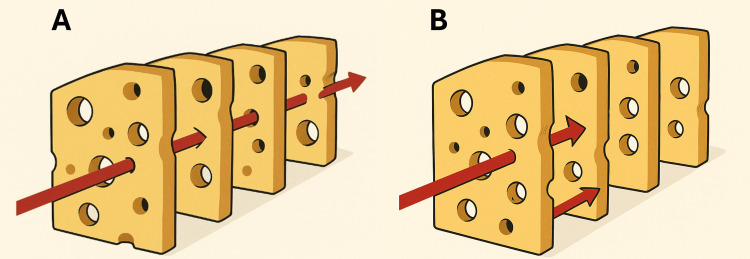
Reason’s Swiss cheese model of system accidents (A) All system elements (defensive layers) align in such a way that an error trajectory (red arrow) passes through each layer’s vulnerabilities, resulting in a system failure. (B) A system layer interrupts the trajectory of the error, effectively preventing the failure by blocking its passage. Image created by using DALL-E (OpenAI, California, US)

Reason’s Swiss cheese model of system accidents posits the following key principles: (1) Every high-tech system comprises multiple layers of defense, each offering a specific level of protection against potential hazards; (2) Analogous to the holes in slices of Swiss cheese, these defense layers exhibit imperfections and vulnerabilities; (3) In a real-world system, these holes are in a constant state of flux, opening, closing, and shifting their positions. An adverse outcome occurs when the holes in each layer momentarily align, creating a trajectory for the opportunity for an accident (Figure 1) [13].

This model illustrates how accidents in complex systems can result from a sequence of events where multiple failures or weaknesses in different layers of defense align to allow an accident to occur. This perspective provides a completely different framework for error management than the traditional person approach, emphasizing systemic factors over individual blame.

Aviation-inspired CRM-type team training programs in healthcare

Since the 1990s, healthcare has drawn substantial inspiration from aviation’s CRM practices, leading to the development of various Clinical or Medical Team Training (CTT/MTT) programs with a common goal of reducing errors and enhancing patient safety (Table 3) [14].

**Table 3:** Medical team training programs in healthcare prior to year 2005 [14]

Simulation-based programs	Classroom-based programs
Anesthesia Crisis Resource Management (ACRM)	MedTeams^TM^
Team-oriented Medical Simulation (TOMS)	Medical Team Management (MTM)
	Lifewings^TM^ (formerly, Dynamic Outcomes Management^©^ )
	Geriatric Interdisciplinary Team Training (GITT)

In the healthcare context, CRM extends to encompass a comprehensive approach targeting situational awareness, decision-making, teamwork, leadership, coping with stress, and managing fatigue, as outlined by Buljac-Samardzic et al. [15]. Team Strategies and Tools to Enhance Performance and Patient Safety (TeamSTEPPS), developed collaboratively by the United States Department of Defense (DoD) and the Agency for Healthcare Research and Quality (AHRQ), was introduced to the public in November 2006 to enhance teamwork skills essential for excellent patient safety. TeamSTEPPS provides a standardized approach that can be tailored depending on the situational context and is based on five domains: structure, leadership, communication, situational monitoring, and mutual support. It has since become the national standard for healthcare team training, representing a pivotal advancement in healthcare safety practices [16].

The foremost step in any successful healthcare team training initiative is to elevate awareness of safety goals and cultivate an environment that fosters the willingness of healthcare professionals to collaborate towards these shared objectives [14]. The body of evidence supporting the effectiveness of CRM-type teamwork training in healthcare system performance now surpasses that in the aviation industry [4]. Healthcare’s slow adoption of CRM-type training may be due to limited data on long-term results, high costs, resource constraints, and the heavy workload of staff. These challenges make it hard to implement large-scale training and show the need for more research and practical solutions.

In a comprehensive systematic review and meta-analysis encompassing English-language literature published between 1985 and 2013, O'Dea and colleagues identified significant outcomes associated with CRM-type teamwork training for healthcare professionals working in acute care settings [4]. Their analysis revealed that such training was well-received by the participating professionals and had a substantial impact on their knowledge and behavior in the short term. However, its effect on their attitudes was comparatively smaller. O'Dea and colleagues recommended further investigation to assess the long-term effects of CRM-type training and its impact on clinical outcomes within the healthcare domain [3].

Another recent systematic review spanning from 2008 to 2018 examined the efficacy of CRM training in comparison to other tools aimed at enhancing team effectiveness within healthcare [13]. This review found wide variation in the content and outcomes of CRM studies. Studies using TeamSTEPPS showed stronger evidence, as they linked specific skills to outcomes. Weaver and colleagues reported that in over 80% of team training studies, participants responded positively, felt more confident, saw better teamwork and safety culture, and noted improved patient outcomes [17].

Future directions and perspectives

Promoting a Culture of Teamwork in Healthcare

In light of the rapid advances in healthcare technologies and the growing demand for specialized professionals in patient care, there is an increasing need for an ever-evolving culture of mutual decision-making, communication, and teamwork (Table 4).

**Table 4:** Key concepts and objectives of healthcare team training

Key concept	Notes
Teamwork mentality and attitude	Teamwork is essential to ensure patient safety, and members should adopt a non-punitive approach to error identification and resolution, refraining from blaming each other [3,14].
Teamwork assessment	Continuous evaluation of team members' attitude, awareness, coordination, and communication is essential [10].
Shared mental model	This provides a critical theoretical model for team building.
Mutual decision making	This should substitute the hierarchical relationship [3].
Inquiry and feedback	Members are encouraged to actively seek comments from other members and adopt a questioning attitude [12].
Backup behavior	During periods of high workload, responsibilities should be smoothly shifted to under-utilized team members [10].
Conflict resolution	This is an important skill that can be acquired through team training and understanding individual team members' approaches to managing conflicts.
Situational awareness	This requires continuous monitoring, information sharing and gathering (through briefing and debriefing), anticipating and planning, and maintaining vigilance [10].
Leadership	Teamwork requires leaders capable of seamlessly overseeing and guiding interpersonal dynamics while setting clear, shared goals.
Adaptability	This is an essential element of teamwork, particularly in an ever-changing healthcare setting filled with daily challenging situations.
Awareness of the communication breakdown	This is probably one of the leading cause of medical errors.
Open and safe atmosphere of communication	Members should feel comfortable speaking up at any time if they have a concern or question, without fear of punishment, humiliation, or intimidation [12].
Mutual respect	No one is allowed to yell, curse, or engage in any form of verbal abuse towards another member of the team.
Mutual trust	Team members should maintain the belief that other members will perform their tasks properly and support each other [10].
Cross monitoring	Team members should be able to identify mistakes and lapses in each other's actions [10].

Overcoming hierarchical divisions stands as a major barrier to progress within healthcare [2]. Building a culture of teamwork requires a system-based approach that promotes: (a) a shared mindset among team members, (b) training to support common goals, (c) regular practice to maintain skills, and (d) ongoing evaluation of team attitudes, coordination, and communication. These elements are key to creating strong, adaptable healthcare teams.

Changing the Culture of Blame in Healthcare

Gordon and colleagues have highlighted the imperative need to replace the prevailing “culture of blame” in healthcare with a culture grounded in mutual respect and shared responsibility [2]. While promoting channels for “error reporting” is essential, it must be done in a manner that does not foster a critical environment, which can adversely impact the morale of healthcare professionals. In the context of a teaching hospital setting, it becomes crucial to closely examine the dynamics between faculties, residents, nurses, technicians, and other staff members. Identifying and addressing poor communication early is key to fostering more positive, productive, and emotionally safe interactions at work. This may require regular involvement of CRM trainers across the hospital departments to observe, assess, and improve communication patterns.

Improving Interprofessional Training in Healthcare Education

Within the current educational paradigm, nurses, physicians, and other healthcare students are typically trained in parallel but separate tracks, often covering similar course content without coordinated instruction. Consequently, meaningful exposure to interprofessional teamwork frequently begins only after graduation. Although some interprofessional training sessions exist, there is growing recognition that such efforts must start earlier in education. Expanding joint training through collaborative assignments across healthcare disciplines can build teamwork skills from the beginning. Integrating this approach into the curriculum helps students develop the attitudes and abilities needed for effective collaboration, ultimately improving patient care and safety.

Standardizing Healthcare Workflow for Comprehensive Patient Care

To develop a comprehensive, team-based approach to patient care, it is imperative to standardize healthcare workflows. Gaps between hospital care and pre- or post-admission care can lead to communication breakdowns that compromise patient safety. To bridge this gap, healthcare teams may need professionals trained to review patient data and coordinate care across settings. These experts can take a holistic approach, improving communication, care continuity, and patient outcomes.

Integrating Approachability Training into Healthcare Team Training

In healthcare team training, teaching approachability is crucial. It means creating an environment where others feel safe to speak up and communicate in ways that suit them. This fosters psychological safety, encouraging open dialogue among colleagues, patients, and families. Training in approachability supports better teamwork, improves care quality, and builds a more inclusive and supportive healthcare environment.

Leveraging AI to Advance CRM Practices in Healthcare

As healthcare evolves, AI and LLMs like GPT are emerging as valuable tools to support CRM-based practices. These technologies offer new ways to improve patient safety, reduce errors, and enhance team communication, the key goals of CRM now being updated for the digital age. One promising use is AI as a real-time communication coach. By analyzing clinical conversations and written communication, AI can detect lapses, such as missed safety checks or breakdowns in closed-loop communication, and provide immediate or post-procedure feedback. These tools also help identify when team members feel unable to speak up, reinforcing CRM’s goal of flattening hierarchies to ensure safety [18].

AI can help amplify the situational awareness, a core CRM concept. By synthesizing data from EMRs, patient monitors, and even communication between team members, AI tools can alert teams to potential blind spots, errors, or cognitive overload. Much like a copilot in aviation who assists the lead pilot in staying aware of system-wide changes, AI systems can support healthcare professionals by providing data-driven prompts and reminders that help them maintain a broader view of the patient’s status [19].

In the realm of simulation and training, LLM-powered platforms offer adaptive scenario generation that responds dynamically to team actions. These simulations move beyond pre-scripted drills to create realistic training experiences tailored to individual team behaviors. Virtual CRM coaches, powered by LLMs, can guide post-scenario debriefs, focusing on non-technical skills like assertiveness, mutual support, and leadership, all of which are integral to safe clinical practice [20].

While thorough notetaking is important, it can sometimes hinder teamwork and efficiency in healthcare. Exploring alternative communication methods and automated documentation can help teams work more effectively. AI tools like LLMs can analyze clinical conversations, generate summaries, highlight team learning points, and track progress, freeing up time for patient care and easing the burden on instructors.

However, integrating AI into CRM-style practices comes with challenges. Teams may become too reliant on AI (automation bias), and there are important ethical concerns around data privacy, especially when analyzing spoken interactions. These issues must be carefully managed to ensure safe and effective use of AI in healthcare teamwork.

If AI tools are trained on biased or incomplete data, they may reinforce unsafe communication patterns or misread subtle human interactions. Still, when thoughtfully integrated, AI, especially LLMs, can act as an intelligent teammate. AI can support CRM principles by improving communication, enhancing situational awareness, and encouraging a proactive focus on patient safety. In this way, AI represents a natural evolution of CRM for modern healthcare [21].

## Conclusions

As we consider the next revolution in healthcare, the key question is whether it will be driven by new treatments or transformative technologies. While both are important, lasting change will require a deeper shift: a reimagining of teamwork, communication, and leadership across the system.

Inspired by the aviation industry and powered by AI, healthcare can adopt proven safety principles of teamwork, clear communication, and strong leadership tailored to its unique context. Unlike airline passengers, patients require care that is not only safe but also deeply personal and morally attuned.

Physicians and healthcare professionals remain committed to putting patients first. By combining this dedication with a renewed focus on team-based care and communication, healthcare can move toward a future where safety, satisfaction, and individualized care define the standard.
